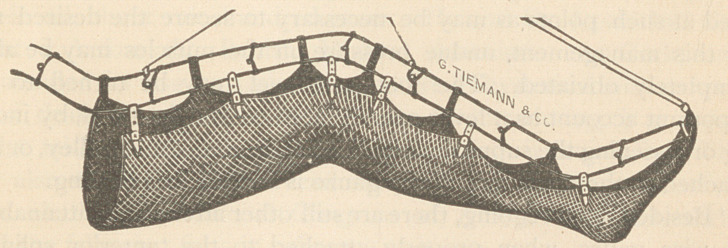# Wire Gauze Supporter for the Treatment of Fractures of the Lower Extremities

**Published:** 1880-02

**Authors:** Harvey L. Byrd

**Affiliations:** Baltimore, Md.


ARTICLE II.

### A WIRE GAUZE SUPPORTER FOR THE TREATMENT OF FRACTURES OF THE LOWER EXTREMITIES.

BY HARVEY L. BYRD, M. D., ETC., BALTIMORE, MD.

The application of this apparatus was first made known to the profession in a paper that was read before the Medico-Chirurgical Faculty of this city, a few years since, in which were set forth the great practical advantages it possesses when attached to the late Professor Smith's "Anterior Splint," or other similar contrivance, in the treatment of fractures and other injuries of the lower extremities. The paper was subsequently published in the Philadelphia *Medical and Surgical Reporter*, and an abstract of it was copied during the same year into the *Half-Yearly Compendium of Medical Science.* A letter was received a short time after its publication, from the distinguished instrument makers of New York city, Messrs. G. Tieman & Co., requesting the privilege of placing the cuts of the supporter among their illustrated instruments, in which they stated that Smith's anterior splint could not be fully utilized without this wire gauze attachment. I now use no other apparatus or appliance than it, in fracture of the lower limbs, and have the greatest reason to be gratified with the success that has attended my cases. Similar experience has followed its use in the hands of other surgeons, and many commendatory letters concerning it have been received from medical gentlemen in different parts of the country. It is so simple, and, as a partial friend has said, "so scientific in its action, that it is only necessary *to be seen to be adopted* by the intelligent practitioner to *the exclusion of every other* appliance in the treatment of fractures and many other injuries of the lower extremities."

It dispenses with the *use of bandages and splints entirely* in the class of injuries for which it was designed and in which it is used, and affords all the comfort to the patient that is attainable in such injuries. Certainly its advantages are vastly superior in *all* respects to *any other*

contrivance thus far known to the profession in all the requisites in fractures of the lower extremities. A number of cases could be added of its successful use since my paper was read before the Faculty, but they would necessarily consume considerable space in the journal; and though such a narrative might prove interesting to some of its readers, it is thought the uses and advantages of the apparatus are so self-evident as not to warrant its being done, and the following from the *Half-Yearly Compendium of Medical Science* will conclude this article:

"Professor Harvey L. Byrd, of Baltimore, describes in the *Medical and Surgical Reporter*, of April 20, a case of injury to the knee-joint, in which he used a support of wire gauze in addition to Smith's anterior splint:

"For this purpose a yard and a half of fine wire guaze was procured, and after taking proper dimensions of the thigh, popliteal region, leg and heel, the gauze was cut so as to adapt it to the posterior and lateral surface of the limb, and then bound around with stout cloth (thin, fine leather has been found, from experience, to answer a better purpose), with buckles and straps attached at suitable intervals. The limb was then made nude by the removal of the bandage that sus-

NOTE.—A A A, is Smith's Anterior Splint, bent to adapt it to the desired flexion of the limb; B B B, is Byrd's Wire Gauze Supporter, applied to the limb and attached to the 'anterior splint;' D represents the suspensory cords and pulley.

tained the splint, and the wire gauze, as above prepared, placed in position under it. When this was accomplished, the 'anterior splint,' receiving proper shape, was placed over the front of the extremity, the wire gauze secured by the buckles and straps to its longitudinal bars, and the hooks from the suspensory apparatus above having been passed into the rings or eyelets of the transverse bars of the 'splints,' the limb was swung from the bed. The greatest comfort was immediately experienced by the patient, and after adjusting the lengths of the straps so as to secure evenness and accuracy of adaptation of the wire gauze to the entire posterior and lateral surface of the limb, the patient was left for the day.

"The whole manipulation, from the time the wire gauze was ready for application until the procedure was accomplished, did not occupy as much time as has been consumed in writing this account of it. If the foregoing details are sufficiently clear of comprehension, it will be observed that the important objects of extension and counter-extension were as fully accomplished in this case as they could have been by any other means, while the great relief from all anterior compression of muscles and the free exposure of the surface of the limb to the atmosphere, not only anteriorly, but laterally and posteriorly, also, was sufficient to impart the great comfort she experienced from the change of treatment. But the scientific surgeon will readily perceive other, and, if possible, greater advantages, which may be obtained from the wire gauze in the management of fractures and wounds of the lower extremities; or, perhaps, we should rather say, great additional advantages; for it is difficult to conceive of any much greater advantage than free ventilation in such cases. The chief of these alluded to, for example, as in compound and comminuted fractures, etc., will be sought for in vain outside of the wire gauze.

"A wound may be clearly seen and freely syringed or sponged, and receive other local treatment through the wire gauze, even when situated on the posterior or lateral aspect of the limb, without disturbing its position in the slightest uncomfortable degree. Again, where it is desirable to remove, at any point, undue, or uncomfortable pressure, nothing is more easily accomplished, as the leather straps and buckles attached to the margin of the wire gauze may be slackened or tightened at such points as may be necessary to secure the desired relief. By this management, undue pressure on the muscles may be almost completely obviated. The foot and heel may be turned to most important account as a lever or weight, in certain fractures, by increasing or lessening the support which the anterior cord and pulley, or sling, attached to the distal end of the gauze is capable of effecting.

" Besides the foregoing, there are still other advantages attainable by the wire gauze, when properly attached to the 'anterior splint,' or other support. But these will readily suggest themselves to the intelligent practitioner in individual cases. The writer has become satisfied, from his own and the experience of friends, that with wire gauze sufficiently fine, accurately cut, and properly adjusted to the limb and anterior support, less sagging takes place than under any other method of treating fractures, and consequently less deformity results from this than any other mode of treatment where bandages are used."

---

ARTICLE III.

## Figures and Tables

**Figure f1:**
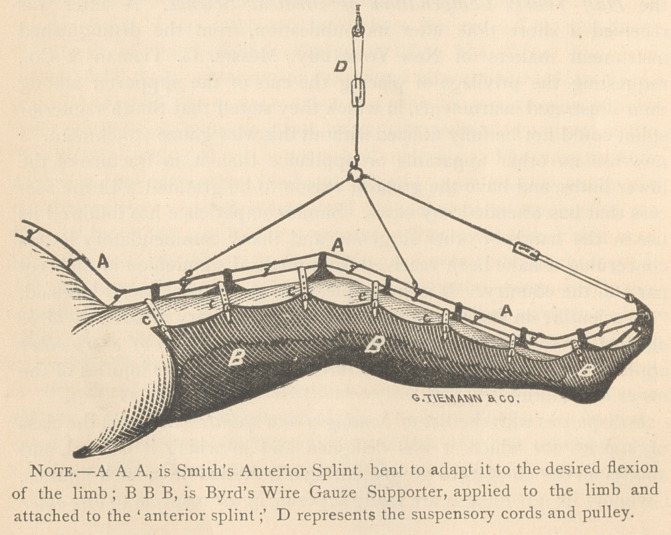


**Figure f2:**